# Curcumin: a calixarene derivative micelle potentiates anti-breast cancer stem cells effects in xenografted, triple-negative breast cancer mouse models

**DOI:** 10.1080/10717544.2017.1381198

**Published:** 2017-09-28

**Authors:** Wei Chen, Lin Li, Xiaofen Zhang, Yuan Liang, Zhijun Pu, Lingfeng Wang, Jingxin Mo

**Affiliations:** a Department of Pharmacy, Affiliated Hospital of Guilin Medical University, Guilin, China;; b Department of Pharmacy, Nanchong Central Hospital, Nanchong, China;; c Department of Pharmacy, the 303rd Hospital of the Chinese People’s Liberation Army, Nanning, China;; d School of Pharmacy, Guilin Medical University, Guilin, China;; e Key Laboratory for Stem Cells and Tissue Engineering, Ministry of Education, Sun Yat-sen University, Guangzhou, China

**Keywords:** Triple-negative breast cancer, phosphorylated calixarene, curcumin, breast cancer stem cells, sustainable release

## Abstract

Triple-negative breast cancer (TNBC) is a heterogeneous and clinically aggressive disease with no approved targeted therapy. Curcumin has shown therapeutic potential against TNBC, but it shows low bioavailability and low efficacy when administered as a free drug. Here we describe a novel vehicle for *in vivo* delivery of curcumin based on the phosphorylated calixarene POCA4C6. Curcumin-loaded POCA4C6 micelles (CPM) were prepared using the thin-film method and they showed a unilamellar structure with an average particle size of 3.86 nm. The micelles showed high curcumin encapsulation efficiency and loading was based on liquid chromatography-tandem mass spectrometry. Studies with cell cultures suggest that CPM can sustainably release curcumin in a pH-dependent manner. The micelles efficiently inhibited proliferation, invasion, migration and tumor spheroid formation by BT-549 human breast cancer cells. These effects involved increased apoptosis and reduced levels of nuclear β-catenin and androgen receptor. After injection into tumor xenografts, CPM persisted in the tumor tissue and efficiently inhibited tumor growth without causing obvious systemic toxicity. CPM also significantly reduced levels of CD44^+^/CD133^+^ breast cancer stem cells. Our results highlight the potential of CPM as an effective therapy against TNBC.

## 1. Introduction

Breast cancer is the most commonly encountered form of cancer and results in high mortality rates. Triple-negative breast cancer (TNBC) is particularly difficult to treat because the malignant tumors lack the receptors for estrogen, progesterone and human epidermal growth factor receptor 2, which are targeted by most available breast cancer chemotherapies (Aceto et al., 2012). TNBC can also be resistant to radiotherapy and it shows relatively high rates of recurrence (Reya et al., 2001). Therapeutic resistance and recurrence may be mediated by low-abundance breast cancer stem cells (BCSCs). Since up-regulation of β-catenin and androgen receptor is associated with TNBC metastasis, recurrence and poor prognosis, inhibiting β-catenin and androgen receptor expression or activity in BCSCs may be effective against TNBC. (Dey et al., 2013; Proverbs-Singh et al., 2015).

Like other natural polyphenols in the diet, curcumin has been shown to reduce the risk of various types of cancers. It appears to inhibit tumor growth via several mechanisms, including arresting the cell cycle, inducing apoptosis, inhibiting angiogenesis as well as multiple pro-survival signaling pathways and down-regulating the expression of anti-apoptotic proteins (Bhattacharyya et al., 2007; Panda et al., 2017). Curcumin is also attractive for drug development because it can be tracked easily *in vitro* and *in vivo* based on its fluorescence emission (Gogoi & Sen Sarma, 2015). However, clinical use of curcumin remains a challenge because of its poor bioavailability and anti-tumor activity. Efforts to improve curcumin stability and bioavailability by delivering it in nanoparticle formulations have shown promise against several cancers, but these approaches are unlikely to be effective against highly heterogeneous, therapeutically challenging TNBC.

**Figure 1.**
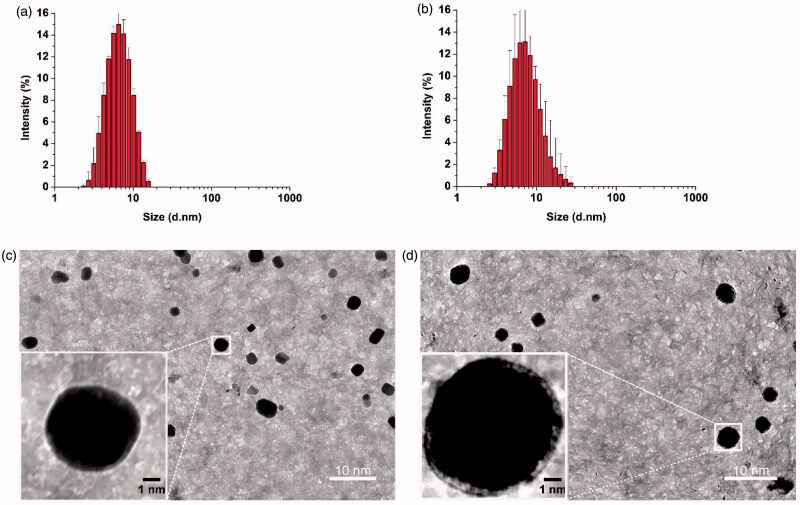
Thin-film method to prepare empty POCA4C6 micelles (PM) and curcumin-loaded POCA4C6 micelles (CPM). Size distributions of (a) PM and (b) CPM based on dynamic light scattering. Transmission electron micrographs of (c) PM and (d) CPM. (Scale bar, 10 nm, 1 nm in inset).

Therefore, we developed a novel nanocarrier for curcumin based on the phosphorylated amphiphilic calixarene POCA4C6, which carries hydrophilic phosphate groups on the upper rim and hydrophobic alkyl groups on the lower rim (Figure 1, inset). POCA4C6 has been shown to self-assemble into vesicles or micelles that can encapsulate drugs and release them in a pH-dependent manner (Mo et al., 2015, 2016). In addition to serving as a drug delivery platform, calixarene itself can show therapeutic activity: calixarene derivatives can inhibit cancer cell proliferation and invasion as well as tumor angiogenesis by inhibiting signaling pathways (Astorgues-Xerri et al., 2014). Using calixarene in combination with some anticancer drugs can provide greater therapeutic efficacy than using the drugs on their own.

In the present study, we explored whether POCA4C6 could act not only as a biocompatible nanomaterial to encapsulate curcumin but also as an adjuvant to enhance curcumin efficacy against TNBC. Curcumin-loaded POCA4C6 micelles (CPM) were prepared by thin-film dispersion and their morphology, encapsulation efficiency and pH-dependent release of curcumin were studied. We assessed the ability of CPM to reduce the viability, cell cycle progression, migration, invasion and sphere formation by BT-549 human breast cancer cells. We also examined the effects of CPM on expression of β-catenin and androgen receptor in these cells. Finally, we injected CPM into human TNBC tumor xenografts in nude mice and examined the micelles *in vivo* distribution, anti-tumor effects and systemic toxicity.

## 2. Materials and methods

### 2.1 Reagents

All reagents, solvents, chemicals and cell culture plastics were obtained from Sigma-Aldrich Co. (St. Louis, MO) or Thermo Fisher (Pittsburgh, PA) unless otherwise mentioned. Annexin V-FITC (fluorescein isothiocyanate)/PI (propidium iodide) apoptosis kits were purchased from Lianke Technology (Hangzhou, China). Phosphonato-calixarene (POCA4C6, purity >95%) was synthesized in our laboratory. Curcumin and doxorubicin were obtained from Aladdin Chemical Reagent (Shanghai, China). Paraformaldehyde (4%) was purchased from Guangzhou Ruishu Biotechnology (Guangzhou, China). Ultrapure deionized water was prepared using a Millipore system with resistivity of 18.2 MΩ.cm.

### 2.2 Cells

Human BT-549 and MCF-7 breast cancer cells were obtained from the American Type Culture Collection (Manassas, VA). Cells were cultured in Dulbecco’s modified Eagle’s medium [DMEM] containing 10% fetal bovine serum [FBS] and 1 mM L-glutamine supplemented with 2% penicillin/streptomycin. Antibiotics were purchased from Thermo Fisher (Eugene, OR).

### 2.3 Animals

Female BALB/c nude mice (18 ± 2 g) were obtained from the Model Animal Research Center of Nanjing University (Nanjing, China) and housed under standard sterile and pathogen-free conditions. All animal experiments were performed following the Principles of Laboratory Animal Care (People's Republic of China) and animal protocols were approved by the Ethics Committee of Guilin Medical University.

### 2.4 Preparation of POCA4C6 micelles and loading with curcumin

POCA4C6 was synthesized as described (Mo et al., 2015), in which the lowest-yield step of formulation (70% yield) was adapted from Dondoni et al. (1997). Briefly, *n-*hexyl groups were added to calix[4]arene in DMF using bromohexane and sodium hydride, formulation was carried out *via* the Duff reactio, and the formulated product was reduced to the corresponding alcohol using sodium borohydride. The alcohol was chlorinated using thionyl chloride, phosphorylated using triethylphosphite and deprotected using bromotrimethylsilane. All subsequent reactions were carried out with nearly quantitative yields. The chemical structure of the resulting POCA4C6 was confirmed by ^1^H NMR (Mercury 400, Varian, Palo Alto, CA; Figure S1).

To prepare POCA4C6 micelles containing curcumin (CPM), 150 mg of POCA4C6 and 50 mg of curcumin were dissolved in 50 mL of chloroform in a 150 mL round-bottom flask. The flask was left on a rotary evaporator (BUCHI, Switzerland) overnight in a 40 °C water bath to eliminate chloroform. The resulting thin film was rehydrated for 30 min with 30 mL of deionized water at 40 °C, after which the suspension was sonicated for 10 min using a probe sonicator (Biologics, Cary, NC) at 80% strength. The solution was passed through a 0.22 μm filter (Millipore, Billerica, MA) to remove insoluble material and CPM were freeze-dried and stored at −20 °C for further experiments. The same procedure was used to prepare empty POCA4C6 micelles.

### 2.5 Physical characterization of empty andcurcumin-loaded micelles

Freeze-dried CPM (10 mg) or empty POCA4C6 micelles (10 mg) were rehydrated in 100 mL of phosphate-buffered saline (PBS) at pH 7.4 at room temperature, the solution was passed through a 0.22 μm filter (Millipore, Billerica, MA) to remove insoluble impurities and the resulting solution was analyzed in terms of particle size, polydispersity index (PDI) and zeta potential using dynamic light scattering (Malvern Nano ZS90, UK). Micelle morphology was examined using transmission electron microscopy (JEOL, Japan). Samples were spotted onto copper grids, allowed to dry in a cool dark areas and examined. Encapsulation efficiency and drug loading of CPM were assayed using liquid chromatography followed by tandem mass spectrometry (see supplementary information).

### 2.6 Curcumin release from CPM *in vitro* at different pH’s

Lyophilized CPM was precisely weighed and dissolved in PBS (pH 7.4) to a final curcumin concentration of 2 μM. An aliquot of this solution (5.0 mL) was dialyzed in tubing with a molecular weight cutoff of 2000 Da (Spectrum Laboratories, Rancho Dominguez, CA) against 95 mL of PBS (pH 7.4) or acetate buffer (pH 5.5), both of which contained 0.5% Tween-80. Dialysis was performed at 37 ± 0. 5 °C under stirring at 100 rpm. Curcumin release from CPM was measured at different pH’s at different time spans using liquid chromatography followed by tandem mass spectrometry. Exposure of dialysate to ambient light was minimized during all procedures.

### 2.7 Cellular uptake of CPM *in vitro*

BT-549 cells in logarithmic growth phase were suspended in 1 mL and cultured in 6-well plates at a density of 5 × 10^5^ cells/mL. Then cells were exposed for 12 h to free curcumin (1 μM), CPM (equivalent to 1 μM curcumin), empty POCA4C6 micelles (5 μM) or no additional treatment (control). Supernatants were aspirated, fresh standard medium was added and cells were further cultured for another 24 h. Then the cells were rinsed three times with PBS, fixed in 4% paraformaldehyde for 15 min and stained with DAPI for 15 min. Fluorescence intensity due to curcumin was measured in one set of wells using confocal microscopy (LSM710, Carl Zeiss, Germany) and in a parallel set of wells using flow cytometry (BD Biosciences, Franklin Lakes, NJ).

### 2.8 Effect of CPM on 3 D tumor spheroids

BT-549 cells were allowed to form 3 D spheroids by seeding them at a density of 5 × 10^3^ cells/100 μL per well in a Corning^®^ 96-Well Ultra-Low-Attachment Microplate and incubating them for several days. The resulting tumor spheroids were treated for 24 h with either free curcumin or CPM, in both cases the final concentration of curcumin was 1 μM. Then supernatants were aspirated, fresh standard medium was added and cells were further cultured for another 24 h. Spheroids were rinsed three times with ice-cold PBS, fixed with 4% paraformaldehyde for 12 h, transferred to glass-bottomed petri dishes and covered with glycerophosphate. Fluorescence due to curcumin was observed using confocal microscopy (LSM710, Carl Zeiss) and quantified using flow cytometry (BD Biosciences, Franklin Lakes, NJ).

In another set of experiments, 3D spheroids were incubated for 24 h in culture medium containing free curcumin (1 μM), CPM (equivalent to 1 μM curcumin), empty POCA4C6 micelles (5 μM) or PBS (control). Then supernatants were aspirated, fresh standard medium was added and cells were further cultured for another 10 days. On day 10, spheroids were photographed using phase-contrast microscopy and their diameters were compared.

### 2.9 Anti-tumor activity of CPM *in vitro*

BT-549 and MCF-7 cells were seeded in 96-well plates at a density of 5 × 10^4^ cells/well in a volume of 100 μL. Solutions (100 μL) containing various concentrations of empty POCA4C6 micelles (63.06 μg/L–630.6 mg/L), curcumin (22.14 μg/L–221.4 mg/L) and CPM (129.48 μg/L–1.29 g/L) were added to the wells. Control wells were left untreated. After 24 h incubation, supernatants were aspirated, fresh standard medium was added and cells were further cultured for another 48 h. MTT was added to each well (20 μL, 5 mg/ml) and absorption at 570 nm was measured according to the instructions from the MTT kit manufacturer (Sigma-Aldrich Co., St. Louis, MO). IC_50_ values were determined from these absorbance measurements.

### 2.10 Effect of CPM on apoptosis *in vitro*

BT-549 and MCF-7 cells were plated in 96-well plates at a density of 5 × 10^4^ cells/well in a volume of 100 μL and then were incubated for 24 h with free curcumin, CPM or empty POCA4C6 micelles (3 μM in all cases). Control wells were left untreated. Supernatants were aspirated, fresh standard medium was addedand cells were re-cultured for another 24 h. Later, the cells were trypsinized, pelleted for 5 min at 300 g, rinsed twice with PBS and resuspended in 500 μL of binding buffer. Annexin V (5 μL, 100 μg/mL) and propidium iodide (PI; 10 μL, 50 μg/mL) were added and the suspension was mixed, incubated in the dark for 15 min. Finally the suspension was analyzed by flow cytometry.

### 2.11 Effect of CPM on the cell cycle *in vitro*

BT-549 and MCF-7 cells were seeded in 6-well plates at a density of 5 × 10^5^ cells/well, and incubated for 24 h in 2 mL of culture medium containing free curcumin, CPM or empty POCA4C6 micelles (3 μM in all cases). Control wells were left untreated. Supernatants were aspirated, fresh standard medium was added and cells were further cultured for another 48 h. The cells were harvested, stained with PI and analyzed by flow cytometry.

### 2.12 Evaluation of combination indexes between CPM and doxorubicin

BT-549 or MCF-7 cells (100 μL) were plated in 96-well plates with appropriate density (5 × 10^4^ cells per well). After incubation overnight, the cells were treated for 48 h with varying doses of doxorubicin alone or with 2 μM CPM. Control cells were left untreated. Cell viability was assayed using the MTT kit as described above. Combination indices (CIs) were determined using CalcuSyn V1.0 software (BIOSOFT). When CI is less than 1, the effect of combination is considered to be synergistic.

### 2.13 Effect of CPM on cell invasion and migration in vitro

In experiments to measure cell invasion, BT-549 cells (5 × 10^4^) in 300 µL serum-free DMEM were seeded into the upper chambers of 24-well cell culture plates with polycarbonate inserts with pore diameters of 8 µm (Millipore, Billerica, MA). After 24 h incubation, 800 µL of DMEM containing 10% serum was added to the lower chamber. Later cells were incubated for 24 h with PBS, empty POCA4C6 micelles, free curcumin or CPM, after which supernatants were aspirated, fresh standard medium was added and cells were re-cultured for another 48 h. Cells that had not penetrated the filter were removed using cotton swabs and cells that had migrated to the lower surface of the filter were counted in five randomly selected fields at 40 × magnification (Olympus, Japan). Each assay was performed in triplicate.

In experiments to measure cell migration, BT-549 cells were cultured to confluence for 24 h in 6-well plates in a 5% CO_2_ incubator. The cell monolayer was scratched with a 200 µL pipette tip, then washed twice in PBS to remove floating cells. Cells were then cultured for 6 h in DMEM containing 0.5% FBS in the presence of empty POCA4C6 micelles, free curcumin, CPM or no addition. Supernatants were aspirated, fresh standard medium was added and cells were further cultured for another 18 h. Cells migrating from the leading edge were photographed under a light microscope. The relative gap ratio was calculated using the equation:

$$Relative\;gap\;ratio\;\left(\%\right)=\frac{average\;gap\;distance\;after\;treatment}{average\;gap\;distance\;before\;treatment}\times 100\%$$

### 2.14 Androgen receptor and β-catenin protein extraction and immunoblotting

Nuclear or cytoplasmic proteins were isolated using a CelLytic™ NuCLEAR™ Extraction Kit (Merck, Darmstadt, Germany) according to the manufacturer’s instructions. Lysates were inactivated by boiling and equal amounts of protein (50 μg) were separated on 10% SDS-PAGE gels and electrophoretically transferred to a polyvinylidene difluoride membrane (Millipore, Bedford, MA). Levels of total protein and target proteins were quantified using the Bicinchoninic Acid Kit (Merck, Kenilworth, NJ) or histone H3 (in the case of nuclear extracts) and β-actin (cytoplasmic extracts). The membrane was blocked with 5% dry milk and incubated overnight at 4 °C with primary antibodies against the following proteins: p-AR (1:1000 dilution; sc-52894, Santa Cruz Biotechnology), β-catenin (1:5000; ab32572, Abcam), histone H3 (1:3000; H9289, Merck), and β-actin (1:5,000; A1978, Merck). Membranes were washed with tris-buffered saline containing 0.1% Tween, incubated for 1–2 h with a secondary antibody, then analyzed by enhanced chemiluminescence (Millipore, Billerica, MA).

### 2.15 Tissue distribution of CPM after intra-tumor injection *in vivo*

Female BALB/c nude mice aged 5–6 weeks were purchased from the Model Animal Research Center of Nanjing University (Nanjing, China). BT-549 cells (2 × 10^6^, 100 µL of PBS) were injected subcutaneously on the upper right thigh of mice. When tumors reached a diameter of ∼5 mm, mice were intratumorally injected with 50 μg curcumin in 100 μL saline, the same amount of curcumin in CPM (292.4 μg) in 100 μL saline, 0.5 mg of empty POCA4C6 micelles in 100 μL saline, or 0.9% saline. At 24 h later, *in vivo* fluorescence imaging was performed using a PerkinElmer IVIS instrument (Waltham, MA) with excitation at 442 nm and emission at 530 nm. Subsequently, mice were sacrificed and the tumor, heart, liver, spleen, lung, kidney and intestine were imaged *ex vivo* using the same IVIS instrument.

### 2.16 Effect of CPM on tumor xenograft growth and histology

Mice with tumors of 200 mm^3^ were randomly allocated into four groups (five animals per group) and injected intratumorally with 5 mg/kg curcumin, 29.2 mg/kg CPM, 25 mg/kg empty POCA4C6 micelles or saline once every other day. Animal weights and tumor volumes were measured, with volume defined as 0.5 × long axis (mm) × (short axis (mm))^2^. In accordance with institutional animal care guidelines, animals were euthanized with pentobarbital if tumors reached 1500 mm^3^.

On day 14 after the beginning of intratumoral injections, mice were euthanized, tumors were excisedand weights were recorded. Tumors, brains, hearts, livers, spleens, lungs and kidneys were collected, fixed in formalin and embedded in paraffin and stained with hematoxylin and eosin (H&E). In addition, blood was collected from a postcaval vein, and serum was obtained by centrifugation for 10 min at 1006 g and analyzed using an autoanalyzer (CDC Technologies, Dayton, OH).

### 2.17 Effect of CPM on apoptosis and breast cancer stem cells in tumor xenografts

Tumors treated and excised as described in section 2.16 were cut into slices of 5.0 μm thick, rinsed with PBS three times, incubated in binding buffer and stained with annexin V and PI for 15 min at 25 °C under low-light conditions. Samples were then washed again, stained with 5 mM DAPI and analyzed under a confocal microscope. Tumor slices were also subjected to antigen retrieval in a pressurized heating chamber (Pascal, Dako, Denmark) in tris-EDTA (pH 9) at 115 °C for 60 s before incubation with primary antibodies against CD133 and CD44.

### 2.18 Statistical analysis

Differences were assessed for significance using Student's *t* test between two treatment groups or one-way analysis of variance among multiple groups. Three significance levels were reported: *p* < .05, .01 and .001.

## 3. Results

### 3.1 Physical characterization of CPM and empty POCA4C6 micelles

Based on dynamic light scattering, the average size of empty POCA4C6 micelles at room temperature was 3.10 ± 0.24 nm (Figure 1(a)) and encapsulation of curcumin generated CPM with an average size of 3.86 ± 0.32 nm (Figure 1(b)). Empty POCA4C6 micelles showed a zeta potential of −29.21 ± 5.22 mV, while CPM showed a potential of −25.18 ± 5.74 mV (Table 1). The polydispersity index was well below 0.3 for empty POCA4C6 micelles (0.114 ± 0.046) and for CPM (0.125 ± 0.078), indicating a narrow size distribution in both cases. Transmission electron microscopy showed empty POCA4C6 micelles and CPM to be homogeneous and uniformly spherical (Figure 1(c,d)), consistent with the results of dynamic light scattering.

**Table 1.** Characterization of empty POCA4C6 micelles and CPM.

Sample	Diameter, nm^a^	PDI^a^	Zeta potential, mV^a^	EE (%)^b^	DL (%)^b^
Empty micelles	3.10 ± 0.24	0.114 ± 0.046	−29.21 ± 5.22	N.A.	N.A.
CPM	3.86 ± 0.32	0.125 ± 0.078	−25.18 ± 5.74	95.40 ± 4.50	17.10 ± 1.25

DL: drug loading; EE: encapsulation efficiency; PDI: polydispersity index; CPM: curcumin-loaded POCA4C6 micelles.

^a^ Determined by dynamic light scattering.

^b^ Determined by liquid chromatography followed by tandem mass spectrometry.

The curcumin content of CPM was quantified using liquid chromatography-tandem mass spectrometry as described in the supplementary information. The retention time for curcumin was 2.79 min and calibration curves showed excellent linearity over a concentration range from 2 to 1000 ng/mL (*y* = 2.48x + 63.74, *r*^2^ = 0.996) (Figure S2). Intra- and inter-day precision for measuring low, medium and high concentrations of curcumin showed relative standard deviations less than 2%, in compliance with minimum requirements for assays based on liquid chromatography-tandem mass spectrometry. Encapsulation efficiency was 95.40 ± 4.50 and drug loading was 17.10 ± 1.25%, respectively (Table 1).

### 3.2 Release of curcumin from CPM *in vitro*

**Figure 2.**
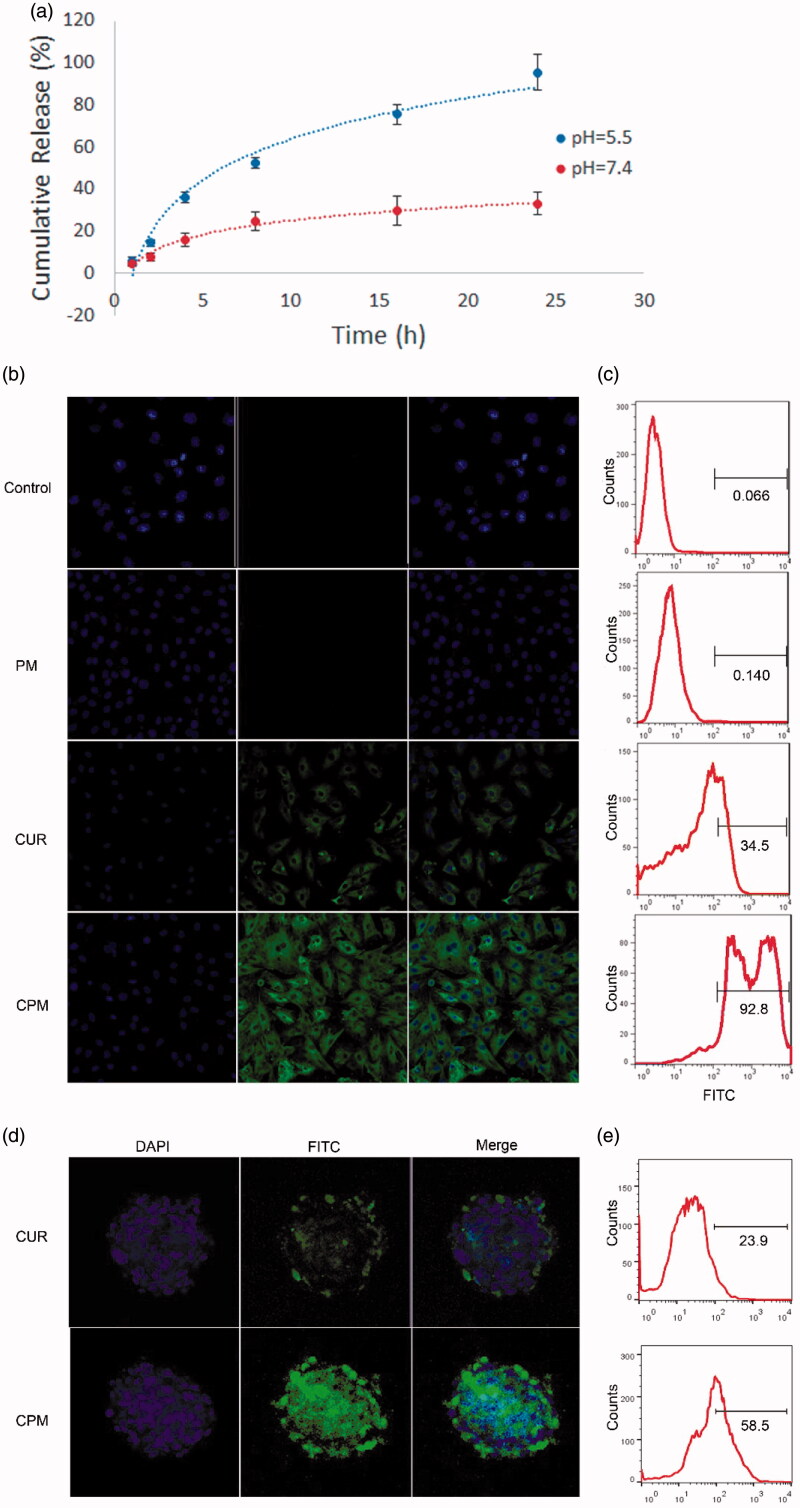
Release profiles and uptake behavior *in vitro*. (a) Fluorescence emission spectra of curcumin release from CPM at pH 7.4 and 5.5, for different time periods from 0 to 24 h. (b) Cellular uptake of empty micelles (PM), free curcumin, or CPM by BT-549 cells (magnification, 200×). (c) Quantitative evaluation of fluorescence intensity in treated BT-549 cells based on flow cytometry. (d) Uptake of PM, curcumin or CPM by BT-549 spheroids (magnification, 160×). (e) Quantitative evaluation of fluorescence intensity in treated BT-549 spheroids using flow cytometry.

At pH 7.4, CPM remained stable, releasing only approximately 30% of curcumin by 24 h at 37 °C (Figure 2(a)). In contrast, at pH 5.5 nearly 50% of curcumin was released from CPM within 8 h and 95% was released by 24 h. These results indicate that CPM can sustain continuous, prolonged release at neutral pH *in vitro*. The faster release at acidic pH likely reflects that the p*K*a_1_ of the four head groups of phosphonate is near 7.2. As a result, these head groups are likely uncharged at pH values well below 7.2, making the micelles less soluble and unstable, therefore accelerating curcumin release.

### 3.3 Uptake of CPM *in vitro*

BT-549 cells were treated with CPM, empty POCA4C6 micelles or free curcumin for 12 h, then nuclei were stained with DAPI (blue) and curcumin was monitored based on its intrinsic green fluorescence using confocal laser-scanning microscopy (Figure 2(b,c)). Cells treated with CPM showed greater cytosolic fluorescence than cells treated with free curcumin (Figure 2(b)), while no significant fluorescence was observed in cells treated with empty POCA4C6 micelles or in untreated control cells. Similar results were obtained using flow cytometry (Figure 2(c)), in which the FITC gating was defined based on cells treated with empty POCA4C6 micelles. The proportion of FITC-positive cells was 92.8% after incubation with CPM, but only 34.5% after incubation with free curcumin. This corresponds to a 269% increase in uptake when curcumin is delivered in CPM.

To complement these experiments with cell monolayers, which may differ from the actual tumor environment, which often shows reduced permeability to drugs, altered enzyme activities and elevated oxygenation, we also performed uptake experiments with 3 D tumor spheroids. Free curcumin appeared to penetrate only the outer layer of spheroids, indicating that deeper diffusion was difficult (Figure 2(d)). In contrast, delivering curcumin as CPM led to its even distribution throughout the entire spheroid. These results were confirmed by quantitating proportions of FITC-positive cells in spheroids using flow cytometry. The proportion of FITC-positive cells was more than two-fold higher in spheroids treated with CPM than in spheroids treated with free curcumin (Figure 2(e)). It is possible that POCA4C6 helped curcumin penetrate into spheroids through membrane fusion, which would be consistent with previous studies (Shi et al., 2013).

### 3.4 Anti-proliferative and cytotoxic effects of CPM in vitro

**Figure 3.**
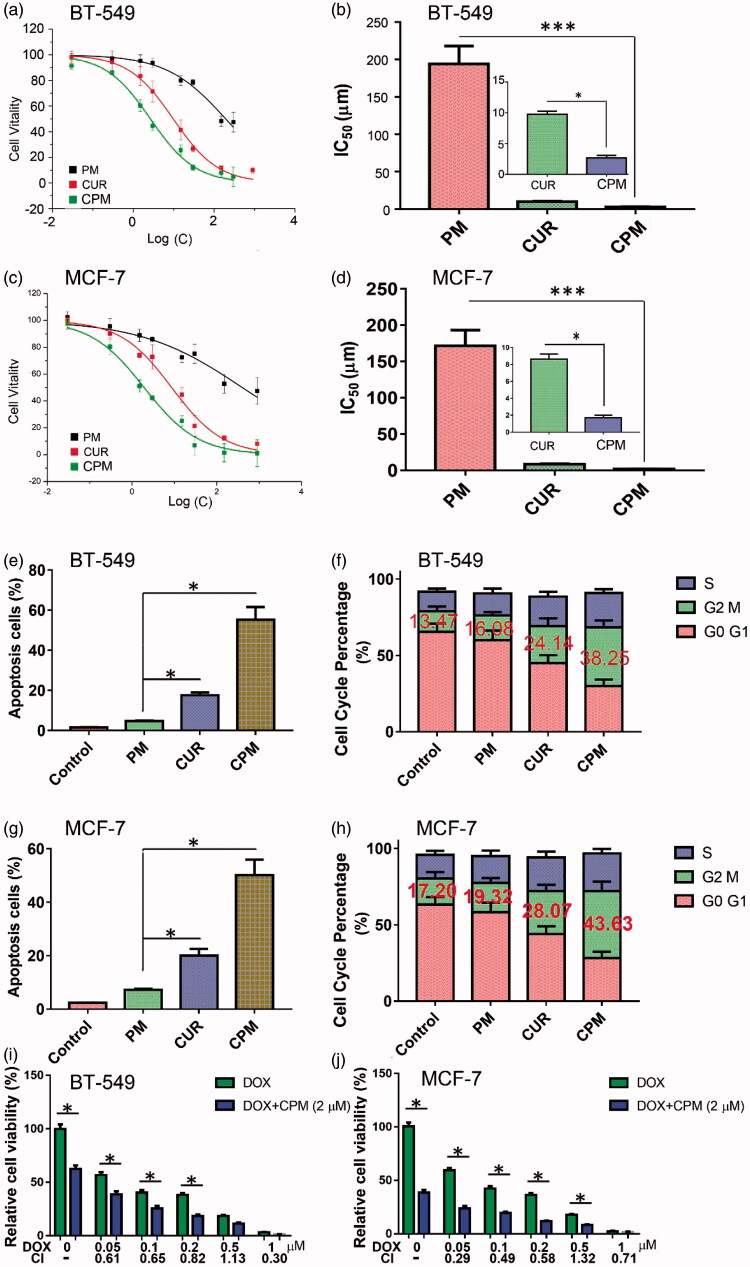
Effects of empty micelles (PM), free curcumin (CM) or CPM on viability, apoptosis and cell cycle of BT-549 and MCF-7 cells. Dose-response curves and IC_50_ values in (a,b) BT-549 and (c,d) MCF-7 cells. Effects on apoptosis in (e) BT-549 and (g) MCF-7 cells. Effects on cell cycle distribution in (f) BT-549 and (h) MCF-7 cells. CPM enhanced the cytotoxicity of doxorubicin (DOX) in (i) BT-549 and (j) MCF-7 cells following 48 h treatment with the indicated DOX concentrations with or without 2 μM CPM. Cell viability was measured using an MTT assay. Combination index (CI) values were determined using CalcuSyn V1.0 software (BIOSOFT).

BT-549 and MCF-7 monolayers were treated for 24 h with empty POCA4C6 micelles, CPM or free curcumin, then incubated another 48 h in fresh culture medium. Proliferation of both cell lines was inhibited in a dose-dependent manner, based on the MTT assay (Figure 3(a,c)), with CPM performing better than the free drug. In BT-549 cells, IC_50_ was 2.67 ± 0.40 for CPM, 9.78 ± 0.51 for free curcumin and 194.35 ± 23.87 μM for POCA4C6 carrier, respectively (Figure 3(b)). Similar trends were observed in MCF-7 cells (Figure 3(d)), where IC_50_ was much higher for curcumin (8.64 μM) than for CPM (1.69 μM). These results suggest not only that CPM can help curcumin enter cells but also that it can synergize with curcumin to slow proliferation.

### 3.5 Effects of CPM on apoptosis and the cell cycle, combination indexes *in vitro*

Treating BT-549 monolayers with CPM containing 3 μM curcumin resulted in 55.3% of apoptotic cells based on flow cytometry, whereas treating them with the same concentration of free curcumin led to only 17.6% of apoptotic cells and treating them with 3 μM POCA4C6 led to 4.7% of apoptotic cells (Figure 3(e)). This corresponds to a 3.14-fold increase in cell apoptosis following treatment with CPM relative to free drug. As shown in Figure 3(g), treating MCF-7 cells with CPM led to 50.2% of apoptotic cells, much higher than the percentages after treatment with curcumin (20.1%) or POCA4C6 (7.2%). The overall analysis indicated that CPM exerted greater pro-apoptotic effects than either carrier or drug on their own (Figure 3(g)).

Cell cycle analysis of BT-549 cells treated with CPM showed that 38.25% were arrested at G2/M phase, significantly more than the 24.14% arrested after treatment with free curcumin (Figure 3(f)) or 13.47% arrested after treatment with empty POCA4C6 micelles (*p* < .05). The same scenario could be observed in MCF-7 cells (Figure 3(h)), where more cells were arrested in G2/M phase when treated with CPM (43.63%) than when treated with curcumin (28.07%) or POCA4C6 (19.32%). These results suggest that CPM exerts anti-proliferative effects by triggering mitotic arrest.

The results in Figure 3(i,j) showed that cell viabilities were much lower after treatment with the combination of CPM and doxorubicin than treatment with doxorubicin alone. CIs for most combinations were far lower than 1.0, indicating synergistic effects on breast cancer cells (Figure 3(i,j)). These results imply that CPM can sensitize the tested cell lines to doxorubicin.

### 3.6 Effects of CPM on invasion, migration, sphere formation and oncogenic signaling *in vitro*

**Figure 4.**
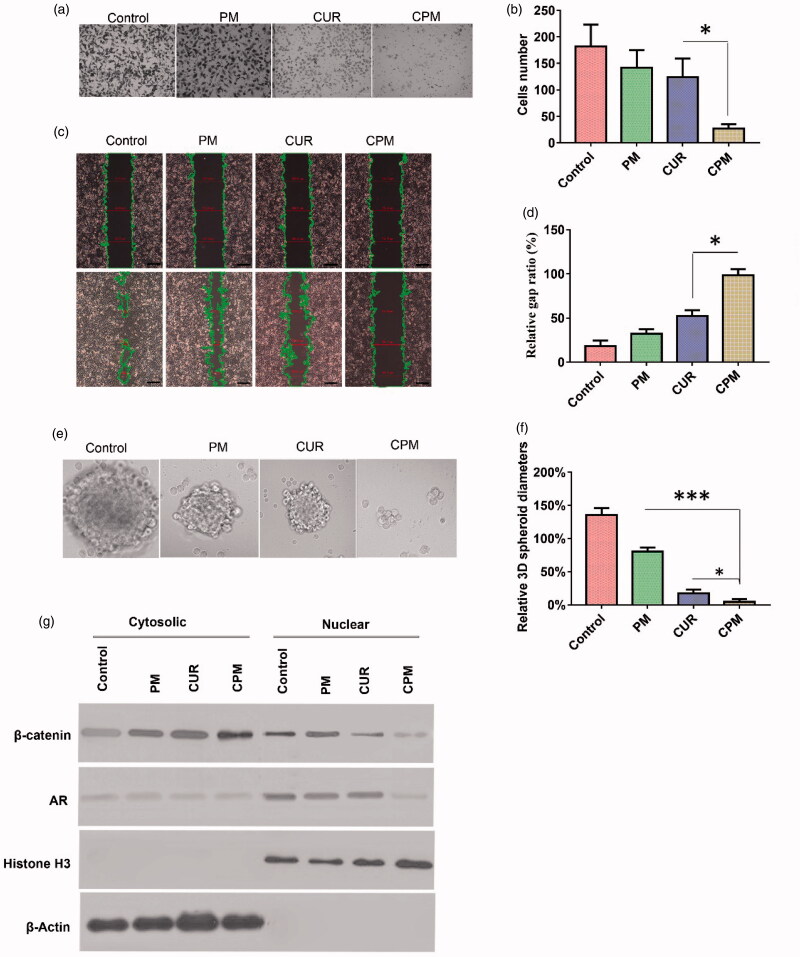
CPM effects on invasion, migration, sphere formation activity, as well as androgen receptor and β-catenin levels in BT-549 cells. (a) Typical images showing inhibition of BT-549 cell invasion by different formulations( magnification, 40×). (b) Quantitation of BT-549 cell invasion. (c) Cells were scratched with a pipette tip, photographed (0 h), then treated with empty micelles (PM), free curcumin or CPM. The control group was left untreated. After incubation in fresh culture medium for another 18 h, migration was photographed under phase-contrast microscopy (Scale bar, 100 μm). (d) Quantitation of gap distance. Ratios of the gap distance relative to the gap at 0 h were plotted. (e) Typical micrographs showing morphology of BT-549 spheroids after treatment with various formulations for 10 days (Magnification, 200×). (f) Relative BT-549 spheroid diameter (normalized to diameter on day 1) after treatment with various formulations. (g) CPM reduced levels of androgen receptor and β-catenin in BT-549 cells. Shown are nuclear and cytosolic levels of these and other proteins in BT-549 cells following treatment with PM, curcumin or CPM. Control cells were left untreated. CPM treated cells showed significantly lower levels of nuclear androgen receptor than the other two treatment groups.

Using a Boyden transwell chamber assay, we found that treating BT-549 cells with CPM significantly reduced the percentage of invasive cells (Figure 4(a,b)). Such treatment also reduced the ability of cells in a monolayer to fill the gap created by pipet scratching in a wound healing assay. After 18 h of incubation, the gap was still 98.37 ± 6.97% of the original width in cultures treated with CPM, compared to 52.34 ± 6.64% in cultures treated with free curcumin and 32.14 ± 5.19% in cultures treated with empty POCA4C6 micelles (Figure 4(c,d)). The gap was nearly completely covered in control cells left untreated.

CPM inhibited the self-renewal ability of BT-549 cells based on a sphere formation assay (Figure 4(e,f)). Tumor spheroids were treated with free curcumin, CPM or empty POCA4C6 micelles, or they were left untreated and then they were cultured for 10 days in standard medium. During this 10 day culturing, tumor spheroid diameter increased approximately by 150% in control cultures, whereas it decreased by 85% in cultures treated by CPM, by 72% in cultures treated with free curcumin and by 35% in cultures treated with empty POCA4C6 micelles.

In order to determine whether these effects on cell migration, invasion and sphere formation may reflect alterations in oncogenic signaling, we examined the possible effects of CPM on expression of androgen receptor and β-catenin. The androgen receptor translocates to the nucleus, where it interacts with β-catenin to trigger gene expression linked to breast cancer. Consistent with previous work showing that curcumin suppresses androgen receptor expression in breast cancer cells, we found that free curcumin down-regulated its expression in BT-549 monolayers. Interestingly, delivering curcumin in CPM led to even stronger down-regulation (Figure 4(g)). Consistent with these results, we found that CPM treatment led to much lower levels of androgen receptor and β-catenin in the nucleus and to higher levels of β-catenin on the cell membrane (Figure 4(g)).

These results suggest that at least some of the observed effects of CPM on BT-549 cell migration, invasion, sphere formation activity and stemness are due to the ability of curcumin to inhibit androgen receptor and β-catenin in the nucleus. This suggests that CPM may be effective against TNBC, since several studies have indicated the therapeutic potential of reducing tumorigenesis in TNBC by targeting nuclear androgen receptor and β-catenin (Pakula et al., 2017).

### 3.7 Results of *in vivo* and *ex vivo* imaging

**Figure 5.**
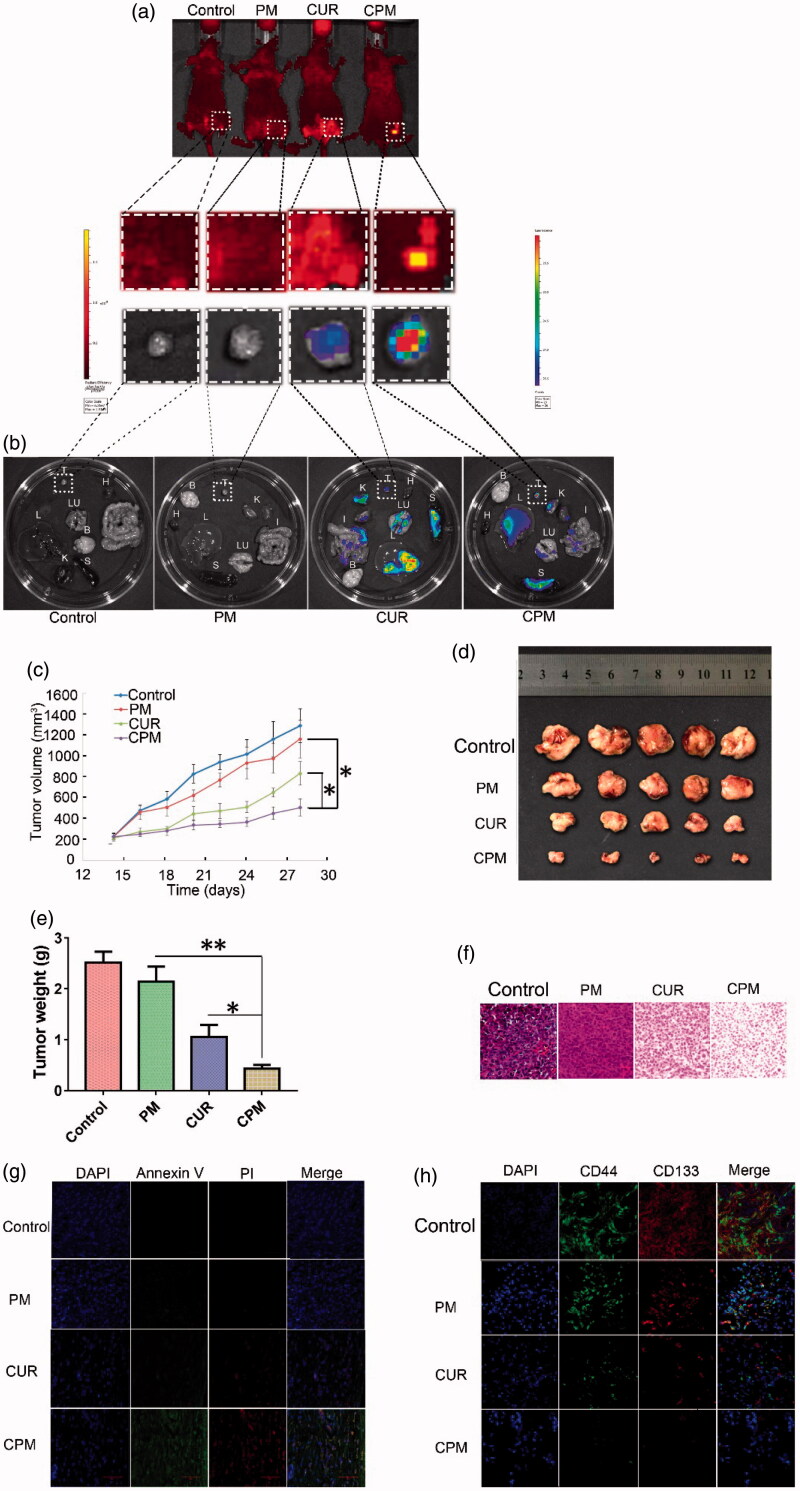
*Ex vivo* fluorescent images and *in vivo* anti-tumor efficacy. CPM preferably accumulated in TNBC xenografts in mice. (a) CPM accumulated at the tumor site at 24 h after intratumoral injection. Curcumin fluorescence was detected using the PerkinElmer IVIS instrument. (b) Empty micelles (PM) and free curcumin accumulated less efficiently in the tumor than CPM. CPM accumulated specifically in the tumor as shown by *ex vivo* imaging of organs harvested at 24 h after intratumoral injection. Images show fluorescence intensity overlaid on the tissue; the corresponding intensity scale is shown at the side of each image. B: brain; H: heart; I: intestine; L: liver; Lu: lung; K: kidney; S: spleen; T: tumor. Anti-tumor capacity of different formulations in BALB/c nude mice bearing BT-549 human breast cancer xenografts. (c) Tumor volumes of mice treated with PM, free curcumin or CPM. Negative control animals were treated with 0.9% saline. (d) Images of the corresponding BT-549 tumors. (e) Weight of excised BT-549 tumors in different groups after treatment. (f) H&E staining of tumor tissues from mice on day 14 after treatment( magnification, 20×). (g) Staining of early apoptosis (annexin V) and late apoptosis (PI) in tumors collected on day 14. (h) Immunohistochemical staining of CD44 and CD133 in tumors from mice after different treatments. CPM reduced expression of both markers (Magnification, 20×).

Epi-fluorescence detection of mice bearing BT-549 tumors was performed to investigate release profiles of the different formulations *in vivo*. CPM showed enhanced sustainable release around the tumor mass at 24 h after intratumoral injection (Figure 5(a)), indicating CPM can accumulate efficiently at the tumor site. Next, tumors as well as other major tissues were excised and imaged *ex vivo* to investigate the systemic distribution of curcumin and CPM. No fluorescence signal was observed in tissues from control mice or mice treated with empty micelles (Figure 5(b)). In animals treated with free curcumin, fluorescence was found in liver, spleen, lung, kidney and intestine as well as in the tumor. Fluorescence was greatest in the liver, followed by spleen. In animals treated with CPM, fluorescence was much greater in tumor than in other tissues, suggesting good tumor-accumulation ability. The distribution in normal tissues showed that CPM was metabolized mainly in liver and kidney, similar to free curcumin, suggesting that both formulations show the same pharmacokinetic profile after entering the circulatory system.

### 3.8 Effect of CPM on breast cancer xenografts *in vivo*

On the basis of the above results, the *in vivo* anti-tumor efficacy of the CPM were further investigated on nude mice bearing BT-549 human breast tumors. Mice were treated with PBS and different drug formulations every 2 days via intratumoral injection and the tumor volume and the body weight were measured every two days (Figure 5(c) and S3a). Tumor growth was suppressed after successive intratumoral injections of empty micelles, curcumin or CPM relative to the growth observed in control animals injected with 0.9% saline (Figure 5(d,e)). It is worth noting that CPM inhibited tumor growth more than the other treatments. Extensive early apoptosis (based on annexin V staining) and late apoptosis (based on PI staining) were observed in tumors treated with CPM, while lower proportions of apoptotic cells were observed in the other groups (Figure 5(g)). H&E staining showed apparent tumor cell necrosis, shrinking and cracking nucleus in tumors treated with CPM, while such effects were nearly non-existent in other groups.

CD44 is a multi-structural and multi-functional cell surface receptor involved in cell proliferation, cell differentiation, cell migration and angiogenesis, while CD133 is believed to be associated with tumorigenicity and progression of TNBC. Both CD44 and CD133 are often used as surface markers of BCSCs (Collina et al., 2015). Therefore, we checked the expression of CD44 and CD133 in cancer cells in tumors after treatment with 0.9% saline, empty micelles, curcumin or CPM *in vivo*. Immunohistochemistry showed reduced expression of CD44 and CD133 following treatment with curcumin or CPM, suggesting that curcumin was effective for destroying CD44^+^CD133^+^ BCSCs. Furthermore, expression of CD44 and CD133 in tumors was minimal after treatment with CPM, suggesting that POCA4C6 and curcumin act synergistically to destroy CD44^+^CD133^+^ BCSCs.

No obvious signs of side effects were observed after treatment with CPM. No deaths or significant loss of body weight occurred following treatment with empty micelles, free curcumin or CPM (Figure S3(a)). Major organs or tissues showed no obvious histopathology based on H&E staining at 28 days after any of the treatments (Figure S3(b)). Hematological indices after treatment with empty micelles, curcumin or CPM were similar to those of control animals (Figure S3(c)). These results suggest that the POCA4C6 nanomaterial shows minimal toxicity *in vivo*.

## 4. Discussion

Here we used the amphiphilic POCA4C6 to create a biocompatible, stable nanoparticle delivery system for curcumin. This molecule is based on work with *p*-sulfonatocalix[4]arene and other aromatic macrocycles showing that such compounds bind selectively and with high affinity to histone trimethyllysine motifs, which are important in gene regulation and oncogenesis (Ali et al., 2015). POCA4C6 features a cavity of the same size as *p*-sulfonatocalix[4]arene, and it has the same number of negative charges. To increase biocompability, the sulfo groups of *p*-sulfonatocalix[4]arene have been substituted with phosphonated groups and four hexane chains have been attached to the lower rim, generating amphiphilic POCA4C6. IC_50_ values of CPM were 2.67 in BT-549 cells and 1.69 μM in MCF-7 cells, much lower than the corresponding values of 194.36 and 171.80 μM for empty POCA4C6 micelles, respectively. Therefore, therapeutic effects of CPM *in vitro* and *in vivo* were superior to those of free curcumin or empty POCA4C6 micelles, suggesting that POCA4C6 can synergize with curcumin. In other words, POCA4C6 may act not only as surfactant but more importantly as adjuvant to potentiate the anti-cancer effects of curcumin.

Curcumin is quite unstable and easily degrades in biological media. Our work shows that encapsulating it into CPM can stabilize it and allow it to exert therapeutic activity over a much longer period and to a greater effect. To ensure accurate analysis of curcumin in our experiments, we used a highly specific liquid chromatography-tandem mass spectrometry procedure involving multiple-reaction monitoring. This avoided false results due to the metabolism of curcumin into various derivatives with similar UV or fluorescence spectra. In addition, our method allows rapid detection, with a retention time of only 2.79 min, as well as high sensitivity, with a low limit of quantitation around 2 ng/mL. This method may prove useful for characterizing curcumin pharmacokinetics in human plasma and for correlating those results with pharmacodynamic endpoints in pre-clinical and clinical settings.

Curcumin is insoluble in water leading to a very poor bioavailability. To prepare free curcumin solution for *in vitro* experiments, it was dissolved in cell culture medium containing 10% dimethyl sulfoxide. The resulting curcumin solution was co-cultured with different cancer cell lines for sufficient time to facilitate diffusion into cells. Then cells were rinsed with fresh PBS to get rid of residual curcumin solution and cultured in fresh cell culture medium, allowing time for the drug to exert its effects. Curcumin is a small, lipophilic compound, so it should easily cross the cell membrane in both directions. Intracellular curcumin may easily exit into the extracellular medium, where it precipitates due to its poor water solubility. If cells are cultured with PCM, these micelles may be taken up by pinocytosis and once inside the cells, they may act as a curcumin depot, from which the drug is sustainably released intracellularly. This may help explain why PCM performed much better than free curcumin in our cell culture assays.

We found that CPM reduced levels of nuclear β-catenin and androgen receptor (Figure 4(g,h)). Disrupting inhibitory signaling mediated by the androgen receptor and disrupting crosstalk between androgen receptor signaling and WNT/β-catenin signaling may be powerful therapeutic strategies for inhibiting breast tumorigenesis (Thakkar et al., 2016). The ability of CPM to shift the distribution of β-catenin from the nucleus to the cell membrane may have important implications for clinical efficacy: analysis of human breast cancer biopsies suggests that high β-catenin levels at the membrane are associated with greater survival probability and lower risk of metastasis than high β-catenin levels in the cytoplasm (Donmez et al., 2016).

Growing evidence implicates BCSCs in breast cancer chemo-resistance and recurrence (He et al., 2016). We found that CPM can efficiently eliminate CD44^+^CD133^+^ BCSCs in BT-549 xenografts and we suggest that this ability depends, at least in part, on the ability of CPM to inhibit signaling by androgen receptor and β-catenin. This implies that CPM can reduce self-renewal and aggressiveness in TNBC. Whether CPM can achieve the same in other cancers should be investigated, since CD44 is not a marker of all cancer stem cells (Pakula et al., 2017).

## 5. Conclusion

We have generated curcumin-loaded POCA4C6 micelles (CPM) using the thin-film dispersion method. CPM has a typical shell-core structure, it forms small particles (3.86 ± 0.32 nm) with a narrow size distribution (PDI, 0.125 ± 0.078) and it can be loaded efficiently with drug (loading ratio, 17.10 ± 1.25%). CPM releases its drug cargo in a pH-dependent manner and this release can efficiently inhibit TNBC both *in vitro* and *in vivo*. CPM facilitates the ability of curcumin to trigger cell cycle arrest, promote apoptosis, inhibit the nuclear activity of androgen receptor and β-catenin and destroy BCSCs. These effects translate to strong ability to inhibit the growth of BT-549 tumor xenografts in mice, without causing obvious adverse effects during 14 days of treatment. These results justify further efforts towards the development of CPM as a breast cancer therapy, including more detailed studies of how POCA4C6 and curcumin synergize to inhibit tumor growth and destroy BCSCs.

## Supplementary Material

IDRD_Mo_et_al_Supplemental_Content.docx
