# Supplementary material for: Curcumin: a calixarene derivative micelle potentiates anti-breast cancer stem cells effects in xenografted, triple-negative breast cancer mouse models
Source: Drug Deliv. 2017 Sep 28;24(1):1470–81. doi: 10.1080/10717544.2017.1381198 (PMC8241084; doi:10.1080/10717544.2017.1381198)
Supplement: IDRD_Mo_et_al_Supplemental_Content.docx [file IDRD_A_1381198_SM7425.docx]

**Synthesis of compound POCA4C6**

^1^H NMR (MeOD/CDCl_3_, 500 MHz) for POCA4C6: peak a=6.58 (s, 8H), peak b=4.38, peak b’=3.08 (2d, *^2^J*=12.4 Hz, 2 × 4H), peak c=3.83 (t, ^3^*J*=7.26 Hz, 8H), peak d=3.33 (m, 8H), peak e=2.78 (d, *^2^J*=20.5 Hz, 8H), peak f=1.93 (m, 8H), peak g=1.38 (m, 24H), peak h=0.92 (t, ^3^*J*=7.12 Hz, 12H).


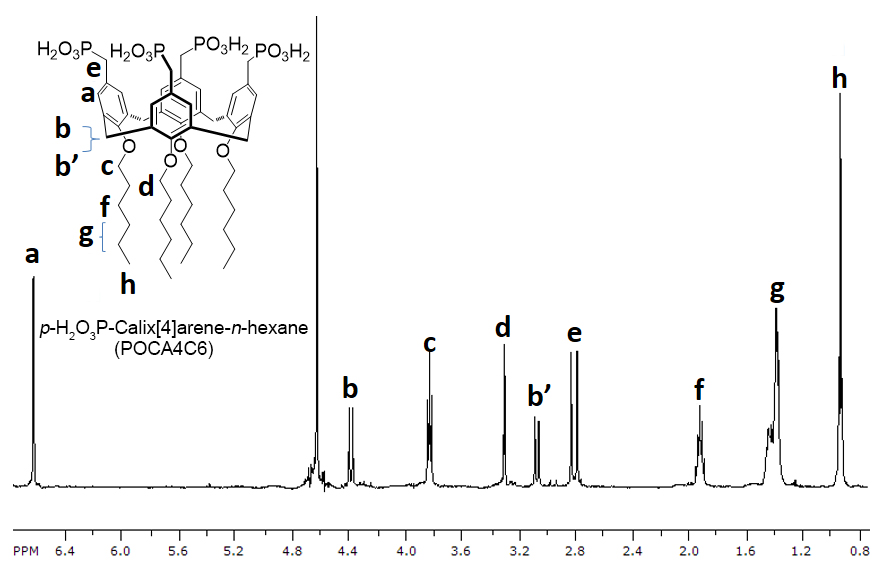


Figure S1. ^1^H NMR spectra for POCA4C6 in MeOD/CDCl_3_.

The POCA4C6 was obtained as white fluffy powders. The purity of POCA4C6 was at least 95 % as determined by HPLC. The ^1^H NMR spectra for POCA4C6 was in agreement with those required by their structures.

**Determination of CPM encapsulation efficiency and drug loading**

Liquid chromatography was performed on a Shimadzu HPLC system (Shimadzu, Columbia, USA). Curcumin and I.S. (hesperetin) were separated on a 50 × 2.5 mm BetaBasic C8 column, 5 μm column (Thermo Hypersil-Keystone, Bellefonte, USA) under an isocratic elution at a flow rate of 0.25 mL/min. The mobile phase consisted of acetonitrile: 0.1 % formic acid solution (50:50 v/v). CUR and the I.S. were monitored using a Finnigan TSQ Quantum EMR Triple Quadrupole mass spectrometer (Thermo Fisher Scientific Corp., San Jose, USA) equipped with an electro-spray ionization (ESI) source. The mass spectrometer was operated in positive ESI mode with a collision gas (Argon) pressure of 1.5 mTorr, a typical electrospray needle voltage of 3000 V, a sheath nitrogen gas flow of 49 (arbitrary unit) and a heated capillary temperature of 350°C. CUR was analyzed by the multiple reaction monitor (MRM) mode using ion transitions at a proper collision energy as follows: CUR m/z 369 > m/z 177 (E = 20 %). The mass spectrometer was tuned to its optimal sensitivity by direct infusion of CUR.

0.5 mg CPM freeze-dried powder was precisely weighed before dissolved in 2 mL of mobile phase. After violently vibration for 2 mins, the supernatant went through 0.22 μm millipore filter before injection for quantification.

Encapsulation efficiency (EE) and drug loading (DL) were calculated by following formula, respectively.

EE (%) = (amount of encapsulated drug / feeding drug) × 100 %.

DL (%) = (amount of encapsulated drug) / (amount of CPM powder) × 100 %

Chemical authenticity of CUR was verified by TSQ quantum triple quadruple mass spectrometer. The full scan mass spectrum (Fig. 3a) of CUR showed a predominant ion at m/z 369.0, resulting from its [M+H]^+^ ion. The [M+H]^+^ ion of CUR was subjected to collision induced dissociation, yielding the most abundant fragment ion at m/z 171.1 (Figure. 3a and b). Therefore, the following transitional channel: m/z 369→171.1 was chose for quantifying CUR.


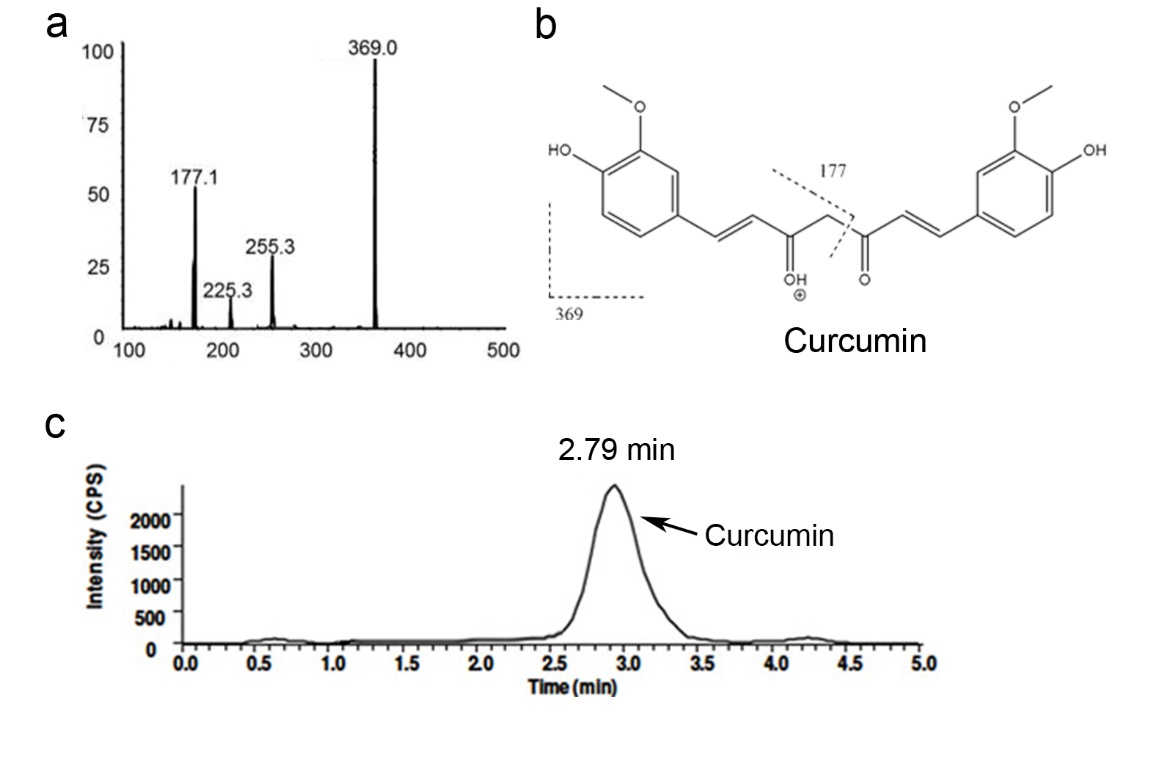


Figure S2. (a) The mass spectra and (b) typical fragments of curcumin (CUR) and (c) its typical chromatograms based on the multiple reaction monitor (MRM).

**Toxicity** **of CPM**


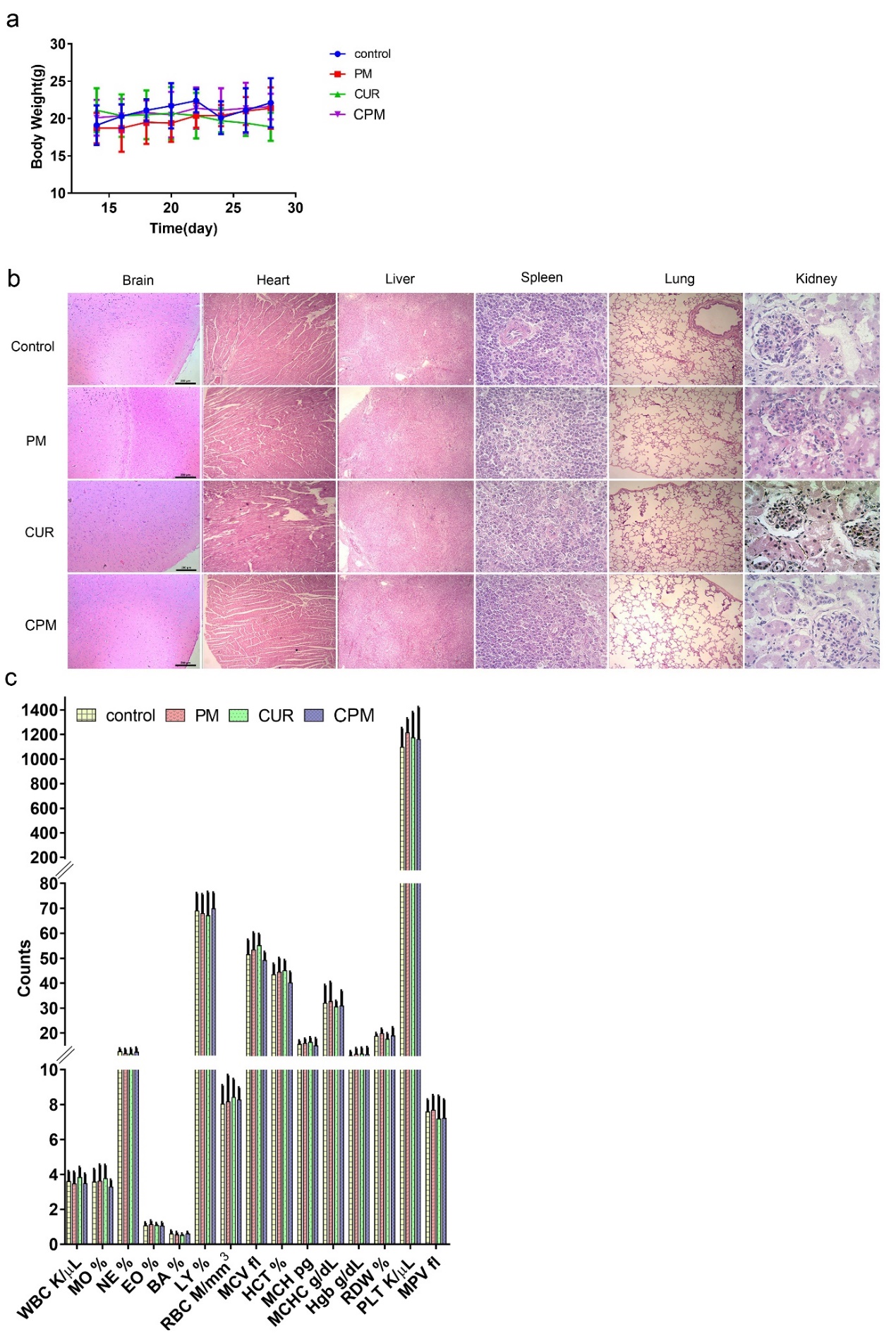


Figure S3. Toxicity of different treatment groups showing no significant systemic damages. (a) Cumulative body weight during treatments. (b) Hematoxylin and eosin (HE) staining of various tissues of mice on the 28th day post inoculation. Scale bar 200 μm (c) Hematological changes in blood of different treatment groups. WBC, white blood cells; LY, lymphocytes; MO, monocytes; NE, neutrophils; EO, eosinophils; BA, basophils; RBC, red blood cells; MCV, mean corpuscular volume; HCT, hematocrit; MCH, mean corpuscular hemoglobin; MCHC, mean corpuscular hemoglobin concentration; Hgb, hemoglobin; RDW, red blood cell distribution width; PLT, platelet; MPV, mean platelet volume. Control group was treated with saline.
